# Yeasts on Tree Gum: Taxonomic, Genetic and Phenotypic Diversity

**DOI:** 10.1111/1758-2229.70393

**Published:** 2026-07-28

**Authors:** Matthias Sipiczki

**Affiliations:** ^1^ Department of Genetics and Applied Microbiology University of Debrecen Debrecen Hungary; ^2^ Institute of Horticultural Science, University of Debrecen Debrecen Hungary

**Keywords:** antagonism, barcoding, diversity, *Metschnikowia*, tree gum, yeast

## Abstract

Depending on their composition, exudates oozing from trees can support or inhibit the growth of microorganisms. Resins released by conifers have a strong antimicrobial effect. Sugar‐rich saps are usually colonised by diverse microorganisms including yeasts. In this study, the yeasts associated with the polysaccharide‐containing gums of cherry trees were examined. 227 strains were isolated from gums of 53 abandoned road‐side and orchard sour cherry trees in 20 locations. 12 ascomycetous and 19 basidiomycetous species were found among the isolates. All grew very poorly on laboratory media containing gum as the only source of nutrients. The high taxonomic diversity and poor growth of the isolates on gum‐based media indicate that the cherry‐tree gum is unlikely to be permanently colonised by a characteristic yeast community. Strains of 16 species showed antifungal antagonism. The *Metschnikowia pulcherrima* isolates killed the cells of the test organisms by releasing pulcherriminic acid. These isolates showed high intragenomic barcode (D1/D2 domain and ITS) diversity due to single‐nucleotide dimorphism (ambiguous nucleotides) in many sites. In the D1/D2 domain, the ambiguous sites were concentrated in four segments that correspond to the back‐folding strands of the stems of the D1 and D2 loops of the secondary structure of the LSU rRNA.

## Graphical abstract

Taxonomically highly diverse yeasts are associated with sour cherry gum, including genetically and physiologically heterogeneous *Metschnikowia pulcherrima* strains that antagonise other fungi by releasing pulcherriminic acid.

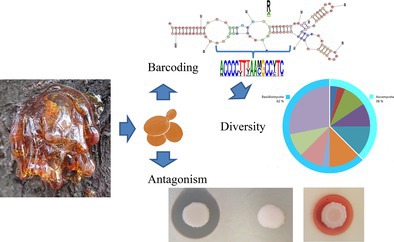

## 1 Introduction

Yeasts are ubiquitous unicellular ascomycetous or basidiomycetous fungi that occur in diverse natural habitats, and are frequently associated with plants and animals. Exudates oozing from certain trees can be colonised by yeasts, provided they contain compounds that can be utilised as carbon sources. For example, the sugar‐rich saps usually host diverse microorganisms, including yeasts (e.g., Lievens et al. 2015; Mushtaq et al. 2004; Péter et al. 2009; Yurkov et al. 2020). Other types of exudates are less suitable for microbial colonisations. Resins produced by conifers are not soluble in water, consist of oleoresin terpenoids, and have an antimicrobial effect against a wide range of microbes (Trapp and Croteau 2001; Savluchinske‐Feio et al. 2006; Rautio et al. 2012). Gums occurring on fruit trees, mainly in the genus *Prunus*, can be less restrictive to microorganisms than resins because they are soluble in water to some extent (for a review, see Barak et al. 2020), and contain high amounts of polysaccharides composed of pentoses and hexoses (Rosik et al. 1966; Olien and Bukovac 1982; Malsawmtluangi et al. 2014).

**FIGURE 1:**
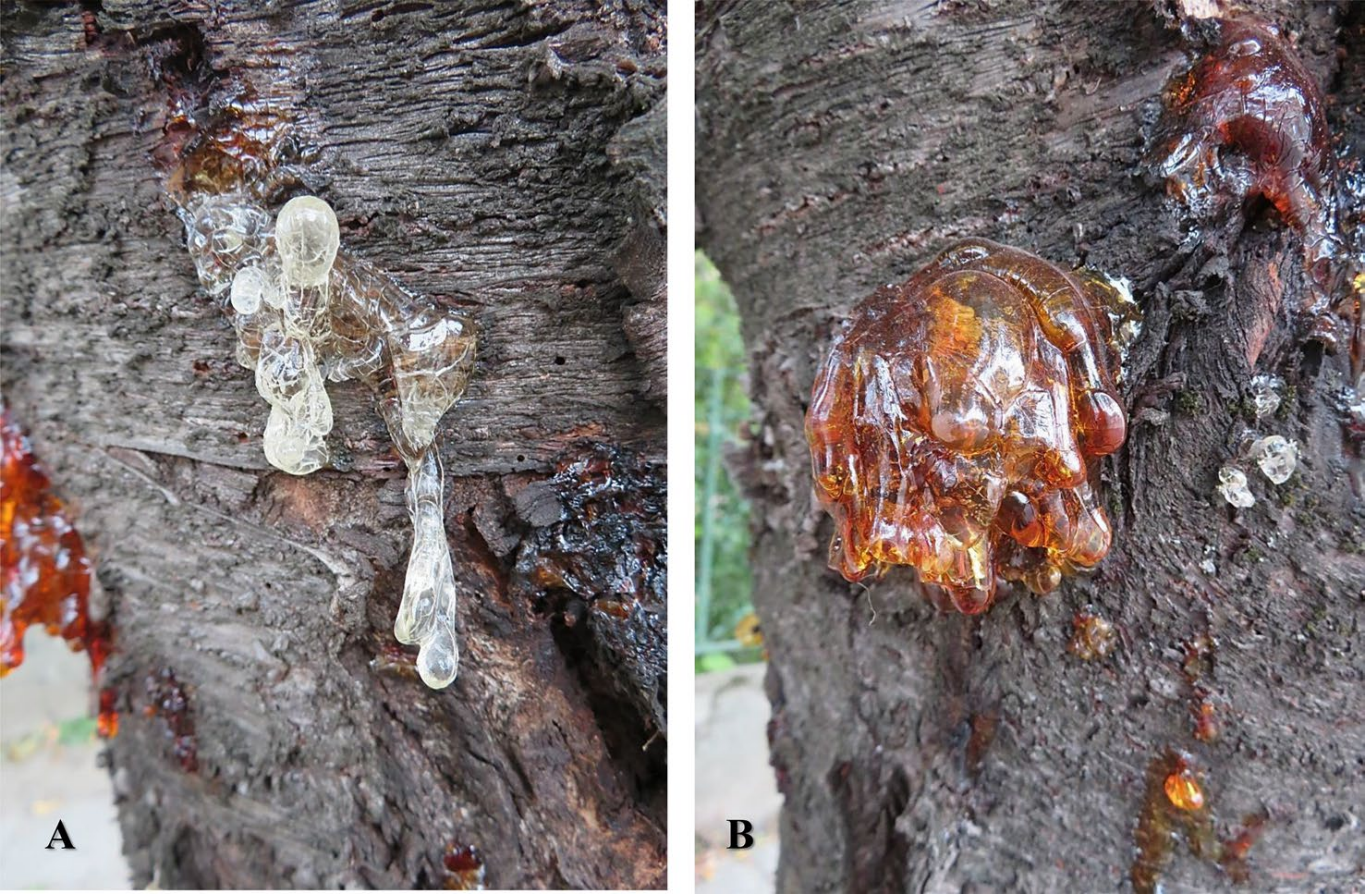
Gummy exudates oozing from the trunk of sour cherry trees. (A) Fresh (soft) gum oozing from the trunk. (B) Mature (hardened) gum.

Gummosis is particularly common on sour and sweet cherry trees. When freshly produced, the oozing gum forms colourless sticky, soft, tear‐like nodules or amorphous lumps that run down branches or trunks (Figure 1A). As time passes, it gradually hardens and turns amber‐coloured (Figure 1B). Gummosis is thought to be a physiological reaction to unfavourable growing conditions (e.g., drought), mechanical or cold injuries (wounds) and is frequently associated with cankers (for a review, see Bouaziz et al. 2016). A canker is a necrotic, often sunken lesion on a stem, branch or twig of the plant that can kill shoots, limb or even the whole tree. Cankers of cherry trees are usually caused by pathogenic fungal species (fungal canker) such as *Cytospora leucostoma* and *Cytospora cincta* (e.g., Barakat and Johnson 1997; Adams et al. 2002) and *Botryosphaeria dothidea* (Ogawa et al. 1995; Weaver 1974) or the bacterium *Pseudomonas syringae* (bacterial canker) (Marroni et al. 2024). By sealing the bark lesions, gums hamper the spread of fungal and bacterial pathogens from the infected tissues on other plants and form a physical barrier against the penetration of further pathogens into the diseased tissues. In addition to serving as a physical barrier, the gum may also antagonise the growth of microorganisms. In a recent study, alcoholic extracts of sour cherry gums were found to reduce the growth of certain food‐borne filamentous fungi under laboratory conditions (Tomar et al. 2022).

However, due to its polysaccharide content, the gum might be a substrate for microorganisms that are able to degrade it to monosaccharides. Recent metabarcoding analyses identified barcode sequences of a very high number of microbial species in gum samples collected from sweet cherry ( *Prunus avium* ) trees (Zhou et al. 2024), suggesting that the cherry gum can be colonised by many microorganisms. However, when interpreting metagenomic and metabarcoding results, it should be borne in mind that these analyses tend to overestimate the taxonomic diversity because they cannot filter out confounding factors such as the intragenomic diversity of barcodes (e.g., Pan et al. 2023; Sipiczki 2022a; Sipiczki 2022b), pollution of the samples with microbes from neighbouring habitats, or with DNA left over from dead cells of previous communities (Brandt et al. 2020), etc. The results of a metagenomic/metabarcoding analysis could be verified by isolating live organisms from the samples, but this is technically unrealistic due to the high number of detected species, and the fact that not all of them can be cultured under laboratory conditions. Live cultures are also needed to study the properties of the individual microorganisms that make up the microbiota.

In order to explore the taxonomic and biological diversity of live yeasts occurring on gums, samples were collected in this study from sour cherry ( *Prunus cerasus* ) gums in different locations. Yeasts were isolated from the samples and representatives of the predominating strains were selected for taxonomic identification, the investigation of physiological properties thought to be relevant for colonisation of the gum and antagonistic interactions with other microbes. This approach cannot explore the entire diversity of the yeast biota because it only considers the most abundant strains. However, it provides a possibility to explore the factors that shape the composition of yeast populations on the gum and their involvement in the protection of the injured plant tissues against pathogens. 12 ascomycetous and 19 basidiomycetous species were detected among the isolates but all utilised the gum poorly as a substrate. The high taxonomic diversity and poor growth of the isolates on gum‐based media indicate that the gum is unlikely to be permanently colonised by yeasts. Their presence is due to external (random) contamination rather than to colonisation. One of the yeast species found in 40% of the samples, *Metschnikowia pulcherrima*, showed high intragenomic and intergenomic barcode and RAPD diversity, physiological heterogeneity, and antifungal antagonism. Strains of another 15 species also had antifungal activities. These yeasts may potentially contribute to the protection of the plant by the gum against pathogen invasions.

## 2 Materials and Methods

### 2.1 Sampling and Isolation of Yeasts

Samples were cut aseptically from amber‐coloured (‘mature’) gums with a sterile scalpel in 20 locations (Table S1). After soaking overnight in YEL (0.5% yeast extract and 2% glucose), loopful amounts of the homogenised material were spread onto YEA (YEL supplemented with 2% agar) plates. After 7 days of incubation at 25°C, 1–2 colonies of each morphological type were isolated from each sample and stored at −70°C.

### 2.2 Barcode Amplification, Sequencing and Sequence Analysis

DNA was isolated from overnight cultures grown in YEL broth and the isolated DNA was used for amplification of barcode segments of chromosomal repeats coding for ribosomal RNAs. The D1/D2 domains and the ITS1‐5.8S‐ITS2 segments were amplified with the primer pairs NL1‐NL4 (O'Donnell 1993) and ITS1‐ITS4 (White et al. 1990), respectively. The same primer pairs were used for sequencing the amplicons in both directions.

The barcode sequences were compared to type‐strain barcode sequences by performing similarity searches in the INSDC (International Nucleotide Sequence Database Collaboration) databases using the NCBI Blast service (https://blast.ncbi.nlm.nih.gov/Blast.cgi). The sequences that differed from all type‐strain sequences available in the databases or were equally similar to more than one type‐strain sequence were deposited in GenBank (http://www.ncbi.nlm.nih.gov/WebSub/?tool=genbank) under accession numbers listed in Table S2.

Multiple sequence alignments were generated with the MUSCLE 3.8.31 algorithm (Edgar 2004). Sequence logos were created from the alignments for each variable region of the D1 and D2 domains with the web‐logo generator available at https://weblogo.berkeley.edu/logo.cgi. Trees were built from the alignments of concatenated variable segments using the neighbour‐joining tool available in the Phylip version 3.67 software package (Felsenstein 2007). Trees were visualised using the FigTree (https://evomics.org/resources/software/molecular‐evolution‐software/figtree/) programme.

Minimum free energy RNA secondary structures were generated for the D1/D2 loops of the cloned allele U45736 of *M. pulcherrima* with Predict, a secondary structure webserver (http://rna.urmc.rochester.edu/RNAstructureWeb/Servers/Predict1/Predict1.html) using default settings.

### 2.3 RAPD‐PCR

Samples of the solutions of genomic DNA (50 ng DNA) were used for RAPD‐PCR reactions with the primers RAPD24 (5′‐GCG TGA CTTG‐3′) (Baleiras Couto et al. 1996) and RAPD1283 (5′‐GCG ATC CCC A‐3′) (Akopyanz et al. 1992) and GoTaq DNA polymerase as described previously (Sipiczki et al. 2024). The amplified fragments were separated in 1.2% agarose gels (1× TBE buffer) and stained with ethidium bromide.

### 2.4 Growth Tests

The ability of the isolates to tolerate high sugar concentrations was tested by dropping 10 μL of cell suspensions (10^7^ cells/mL) onto YEA plates supplemented with various concentrations (20%, 30%, 50% and 60%) of glucose. The ability of the isolates to grow on gum as a substrate, cell suspensions were dropped onto GA (2% grated gum in distilled water), GAN (GA supplemented with 0.5% (NH_4_)_2_SO_4_), GAS (GA supplemented with 2% glucose), and GANS (GA supplemented both with (NH_4_)_2_SO_4_ and glucose). Media with higher amounts of gum were soft or did not solidify (over 10%). Yeast growth was evaluated after 7 days of incubation at 25°C.

To test the isolates for agar penetration, 10‐μL samples of dense cell suspensions (10^7^ cells/mL) were dropped onto YEA plates. After 2 weeks of incubation at 25°C, the colonies were washed off from the plates with water to visualise intrusions in the agar medium beneath them.

### 2.5 Antagonism Tests

The isolates were tested for antimicrobial antagonistic activity with a yeast (*Zygosaccharomyces* aff. *siamensis* 11–2106 (Sipiczki et al. 2024)) and a mould (*Alternaria angustiovoidea* P101; isolated in this study and identified by ITS sequencing) strain as indicator organisms with the “Yeast on indicator (YOI)” and the ‘Confrontation assay on agar medium (CFAA)’ (Sipiczki 2023) methods. In YOI, suspensions of the isolates were dropped on a freshly made lawn of indicator cells on YEA plates. If the isolate has antagonistic activity, a clear inhibition zone (or only a turbid halo) appears in the lawn around its colony. In CFAA, the indicator filamentous fungus was inoculated in the centre of the YEA plate and suspensions of the isolates were dropped at a distance of 2 cm from it. If the isolate has antagonistic activity, it halts the extension of the growing mycelium.

### 2.6 Examination of Pulcherrimin Production

Pulcherrimin production was tested on YEA plates supplemented with 0.02 mg/mL or 0.04 mg/mL FeCl_3_. On these media, the pulcherrimin producers form pigmented colonies (Sipiczki 2020). When pulcherrimin is also produced extracellularly, pigmentation can also be seen in the medium. The isolates were inoculated on these plates by dropping 10 μL of dense suspensions of cells (10^7^ cells/mL). Pigment formation was monitored at 25°C for 4 weeks.

## 3 Results

### 3.1 Culturable Yeasts Isolated From Sour Cherry Tree Gums

Amber‐colour (mature) gums were sampled from 53 abandoned road‐side and orchard sour cherry trees in 20 locations in Hungary in four consecutive years (Table S1). Yeast colonies were formed by all samples when spread onto YEA plates, demonstrating that yeasts culturable under laboratory conditions are regularly associated with gums. For each sample, representatives of all colony types were isolated. Altogether, 227 isolates were collected for taxonomic identification, molecular analysis and phenotypic characterisation.

### 3.2 High Taxonomic (Barcode) Diversity Among the Isolated Yeasts

**FIGURE 2:**
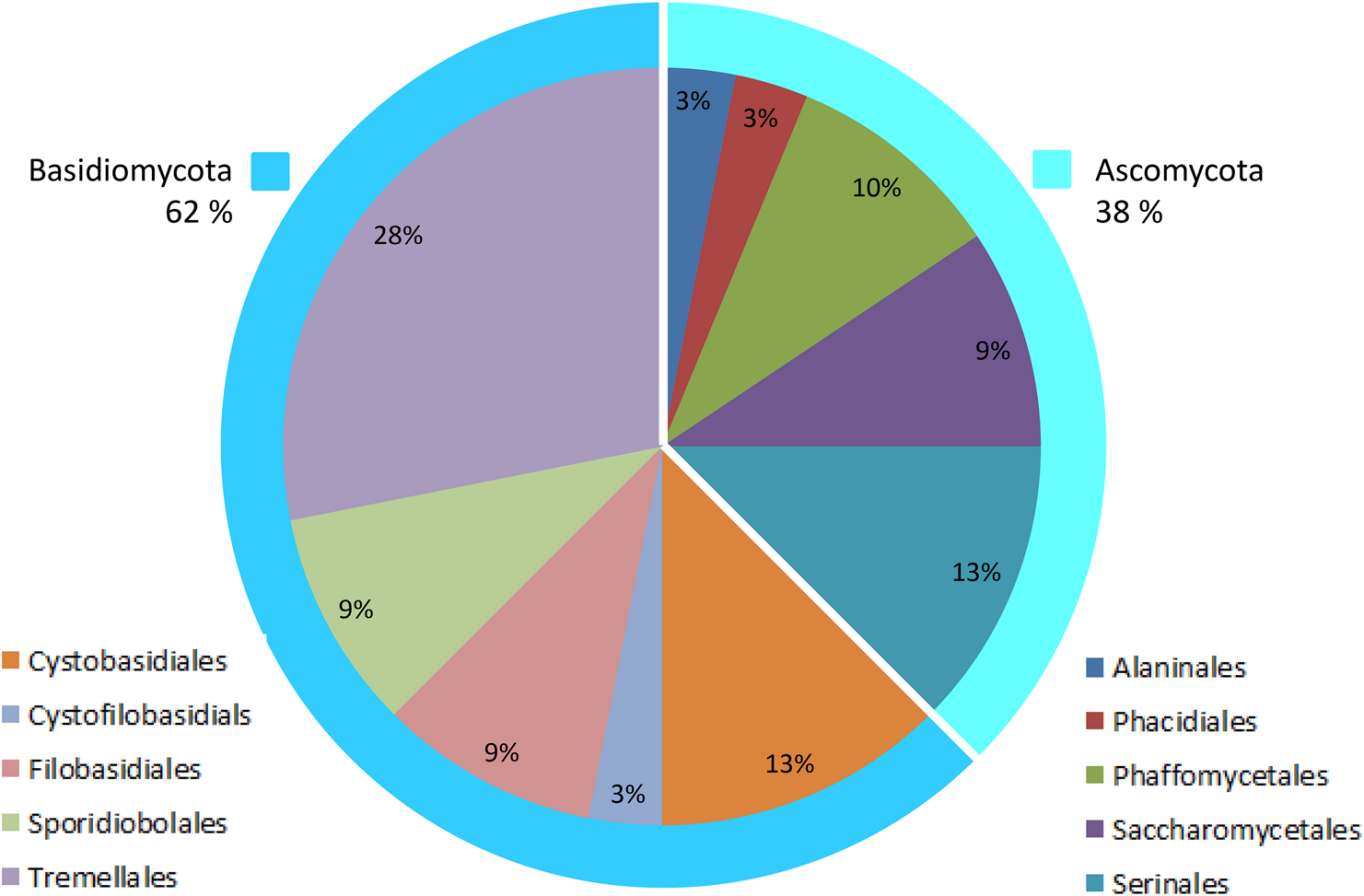
Relative abundance of basodioumycetous and Ascomycetous species among the isolates.

For taxonomic identification, the D1/D2 barcode segments of the rDNA repeats were amplified from each isolate and the amplicons were sequenced. The isolate whose sequences did not differ by more than 1.5% from the most similar type‐strain sequence of a species was considered conspecific with that species. Based on this criterion, 180 isolates could be assigned to known species or at least to known genera (Tables 1 and S2). Figure 2 shows the proportion of the identified basidiomycetous and ascomycetous species and genera by order.

**TABLE 1:** Taxonomic affiliations of isolates.

Taxonomic position	Species	Isolates
**Ascomycota**		
Alaniales	*Nakazawaea holstii*	11–1976, 11–1984, 11–1985, 12–8, 12–30/1, 12–30/2, 12–168/1
Serinales	*Danielozyma* sp. or *Gaillardinia* sp.	12–119, 12–120, 12–121, 12–122
	*Debaryomyces* sp.	12–10, 12–16, 12–17, 12–18, 12–151, 12–154
	*Metschnikowia pulcherrima*	11–1991, 12–7, 12–11, 12–12, 12–13, 12–14, 12–15, 12–117, 12–118, 12–123, 12–124, 12–125, 12–126, 12–127, 12–130, 12–131, 12–132, 12–133, 12–134, 12–135, 12–136, 12–137, 12–152, 12–155, 12–156, 12–158, 12–159, 12–160, 12–161, 12–162, 12–163, 12–164, 12–165, 12–166, 12–167, 12–16, 12–1728, 12–175
	*Meyerozyma guilliermondii*	11–1982
Saccharomycetales	*Lachancea thermotolerans*	12–139
	*Torulaspora delbrueckii*	12–169, 12–170, 12–171, 12–173, 12–174
	*Zygosaccharomyces rouxii*	12–138, 12–140
Phacidiales	*Pallidophorina paarla*	12–26, 12–27, 12–29, 12–170/1
Phaffomycetales	*Wickerhamomyces anomalus*	12–128, 12–129
	*Wickerhamomyces mori*	11–1979, 11–1980
	*Wickerhamomyces silvicola*	11–1983, 11–1994, 11–1995, 11–1996, 12–9,
**Basidiomycota**		
Cystobasidiales	*Cystobasidium psychroaquaticum*	12–19
	*Cystobasidium slooffiae*	12–25
	*Cystofilobasidium infirmominiatum*	11–1963
	*Cystofilobasidium macerans*	11–1971, 11–1974
Cystofilobasidiales	*Vustinia terrae*	11–1981
Filobasidiales	*Filobasidium* sp.	12–23, 12–24
	*Filobasidium wieringae*	11–1973, 12–1, 12–22
Sporidiobolales	*Rhodotorula graminis*	11–1969, 11–1970, 11–1972
	*Sporobolomyces roseus*	11–1968
	*Symmetrospora symmetrica*	12–20, 12–21
Tremellales	*Hannaella surugaensis*	11–1978
	*Kwoniella shivajii*	12–2, 12–6, 12–153
	*Papiliotrema flavescens*	11–1986, 11–1992, 11–1993
	*Pseudotremella hippophaes*	11–1967, 11–1977, 12–4, 12–5, 12–30, 12–157
	*Pseudotremella* sp.	11–1989
	*Teunia* sp.	11–1966, 11–1987, 11–1990
	*Vishniacozyma carnescens*	12–28
	*Vishniakozyma tephrensis*	11–1975, 11–1988
	*Vishniacozyma* sp.	11–1964, 11–1965

The *Debaryomyces* isolates and two *Filobasidium* isolates could not be assigned to single species because their D1/D2 sequences were identical to the type‐strain sequences of more than one species. For these isolates, the ITS barcodes were also sequenced, but neither of these sequences could assign them to one species. The barcode sequences of four groups were very different from all type‐strain sequences available in the INSDC databases. These could only be assigned to genera and are therefore listed in Table 1 without specific epithet. From one tree, strains were isolated whose barcode sequences were separated by almost equal phylogenetic gaps from two genera, *Danyelozyma* and *Gaillardinia*. When two or more isolates from the same sample had identical D1/D2 sequences, one of them was selected for further examination; only these are listed in Tables 1 and S2.

A large group of isolates had ambiguous nucleotides in their D1/D2 sequences. Because of these ambiguities, none of the sequences were identical to any type‐strain sequence. The most similar database sequences were those of *M. pulcherrima* . As different sequences were deposited in the databases for the type strain of this species, multiple similarity values could be calculated for each isolate. Therefore, asterisks were placed into the corresponding cells of Table S2 instead of values. Unfortunately, the sequences of the amplified ITS barcodes were even more heterogeneous (or even incomplete) so that they could not be used to refine the taxonomic affiliation of these isolates.

### 3.3 Intragenomic Barcode Heterogeneity and Intraspecies Genetic Diversity in *Metschnikowia* Isolates

**FIGURE 3:**
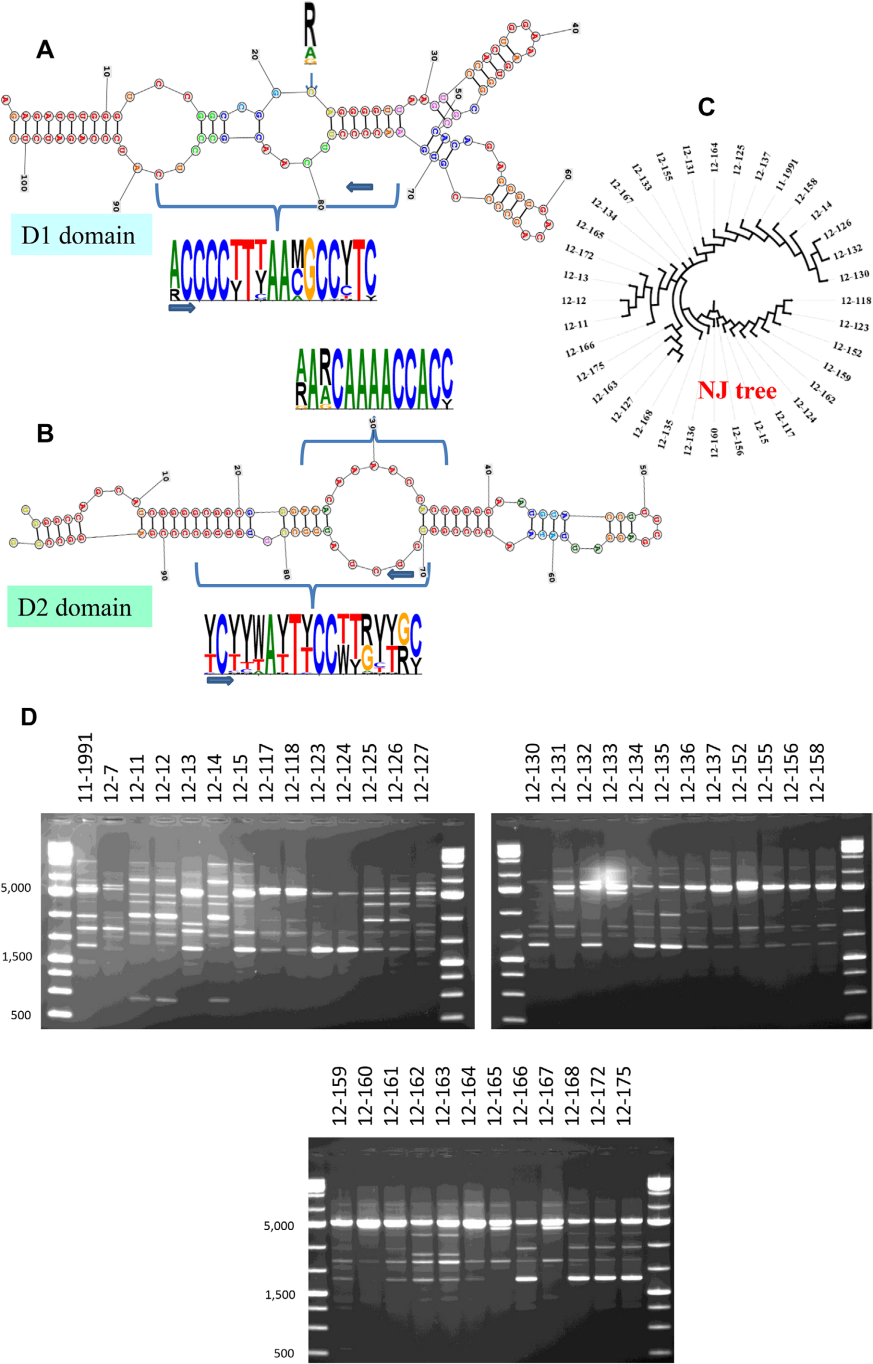
Barcode and RFLP diversity of *M. pulcherrima* isolates. Variable segments shown on the predicted secondary structures of D1 (A) and D2 (B) hairpin–stem loops of the transcript of U45736 of the *M. pulcherrima* type strain. (C) Neighbour‐Joining tree inferred from the alignment of concatenated variable D1/D2 segments of the isolates. (D) RAPD patterns of the isolates.

Ambiguous nucleotides in the D1/D2 and ITS sequences of the *Metschnikowia* isolates indicated that their repeats that code for rRNAs differed in sequence (intragenomic diversity). All ambiguous sites were dimorphic (SND: single nucleotide dimorphisms) where two peaks were seen in the chromatograms. The ambiguous sites of the D1/D2 domains were concentrated in four segments that correspond to the back‐folding strands of the stems of the D1 and D2 loops of the secondary structure of the LSU rRNA (Figure 3A,B). The isolates varied in the number and, to some extent, also in the locations of the variable sites (intergenomic/interstrain diversity). Despite this variability, the D1/D2 sequences did not form clearly separated clusters on the Neighbour Join tree (Figure 3C). Thus, the *Metschnikowia* isolates were conspecific but had diverse chromosomal repeats encoding ribosomal RNAs. Interstrain diversity was also detected when the genomes of the isolates were compared with whole‐genome RAPD analysis (Figure 3D). However, there was no correspondence between the D1/D2 and the RAPD diversity. For example, the isolates 12–136, 12–155, 12–156, and 12–158, which had identical RAPD patterns, sit on very distant branches of the barcode tree.

### 3.4 The Gum Is Unfavourable Environment for Yeasts

The presence of yeasts on the gum samples could be attributed to yeast growth in the cherry tree gum. However, its high polysaccharide content may have an adverse effect on the yeast cells, for example, by creating high osmotic pressure. Unfortunately, this possibility could not be tested in laboratory conditions because the agar media supplemented with more than 10% grated gum did not solidify. Therefore, the isolates were tested for osmotolerance on media supplemented with higher concentrations of glucose. Certain isolates could grow even at 60% glucose (Figure S1).

Then the isolates were tested for growth on plates of media prepared with gum as the only source of nutrients. All isolates grew very poorly on these media. Supplementation of the gum media with glucose or with ammonium sulphate only slightly improved their growth. Significant improvement was achieved by simultaneous supplementation with both compounds. So, the gum is poor in nutrients required by the isolated yeasts for growth.

### 3.5 Substrate Penetration by Ascomycetous Isolates

**FIGURE 4:**
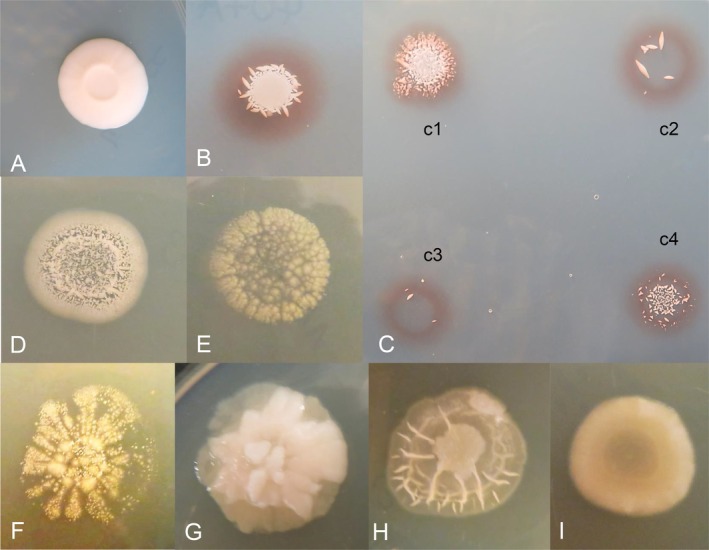
Invasive growth. (A) *M. pulcherrima* 12–165 colony formed on agar medium. (B) Its intrusions into the medium made visible by washing off the colony. (C) Diverse invasivity of *M. pulcherrima* isolates: (c1) 12–127, (c2) 12–134, (c3) 12–159, (c4) 12–160. (D) *Zygosaccharomyces rouxii* 12–138. (E) *Meyerozyma guilliermondii* 11–1982. (F) *Nakazawaea holstii* 11–1976. (G) *Danielozyma* sp. 12–121. (H) *Wickerhamomyces anomalus* 12–128. (I) *Vishniacozyma carnescens* 12–28.

When cultivated on agar plates, all ascomycetous yeasts could grow into the medium (Figure 4A–I, Tables 2 and S3). Penetration was also observed on the gum medium supplemented with glucose and ammonium sulphate but no penetration was visible on non‐supplemented gum media. Except for two species (*Vishniacozyma carnescens* and *Pseudotremella* sp.), the basidiomycetous yeasts failed to grow into the medium. The *Metschnikowia* isolates formed intrusions into the medium with very variable efficiency (examples are shown in Figure 4B,C) (Table S3). It was not possible to quantify the effectiveness of the substrate invasion as the morphology of the intrusions and the density of the invasion sites varied greatly even within species.

**TABLE 2:** Antagonism, synergism and invasive growth.

Species	Effect on the growth of		Invasive growth
	*Zygosaccharomyces* 11–2106	*Alternaria* P101	
**Ascomycota**			
*Danielozyma/Gaillardinia* sp.	−	z/c.a	++
*Debaryomyces* sp.	−	−	−/++
*Lachancea thermotolerans*	−	c	+
*Metschnikowia pulcherrima*	Variable, see Table S3		
*Meyerozyma guilliermondii*	−	z	+++
*Nakazawaea holstii*	−	z,a	−/++/+++
*Pallidophorina paarla*	−	z	+++
*Torulaspora delbrueckii*		−	+
*Wickerhamomyces anomalus*	turbid	z	+++
*Wickerhamomyces mori*	turbid	z,c	+/++
*Wickerhamomyces silvicola*	−/turbid	−/(c),a	−/+++
*Zygosaccharomyces rouxii*	−	−	+++
**Basidiomycota**			
*Cystobasidium psychroaquaticum*	−	−	−
*Cystobasidium slooffiae*	−	−	−
*Cystofilobasidium infirmominiatum*	−	−	(+)
*Cystofilobasidium macerans*	−	−	−
*Filobasidium* sp.	−	z,c	−
*Filobasidium wieringae*	−	−	−
*Hannaella surugaensis*	−	c,a	−
*Kwoniella shivajii*	−	−	(+)
*Papiliotrema flavescens*	−	−/c.a	−
*Pseudotremella hippophaes*	−	−/c,a	(+)
*Pseudotremella* sp.	−	−	+
*Rhodotorula graminis*	−	−	−
*Sporobolomyces roseus*	−	−	−
*Symmetrospora symmetrica*	−	c,a	−
*Teunia* sp.	−	−	−/(+)
*Vishniacozyma carnescens*	−	z	+++
*Vishniakozyma tephrensis*	−	−	−
*Vishniacozyma aff melezitolytica*	−	−	−
*Vustinia terrae*	turbid	(c),a	−

Abbreviations: −: no activity; +: weak activity, ++: medium activity; +++: strong activity; a: the growth of the mycelium is facilitated around the yeast colony (e.g., Figure 5f2); c, contact inhibition; turbid: halo of reduced growth rate; z, clear zone of inhibition.

### 3.6 Inhibition (Antagonism) and Promotion (Synergism) of Growth of Test Microorganisms by Isolated Yeasts

As gumming has been hypothesised to have a protective function against pathogenic microorganisms (e.g., Tomar et al. 2022), the isolates were also tested for antimicrobial activities with a yeast and a filamentous fungus as indicator (test) organisms. As predicted, certain isolates inhibited the growth of the indicator organisms, but somewhat unexpectedly, other isolates facilitated their growth (Table 2).

**FIGURE 5:**
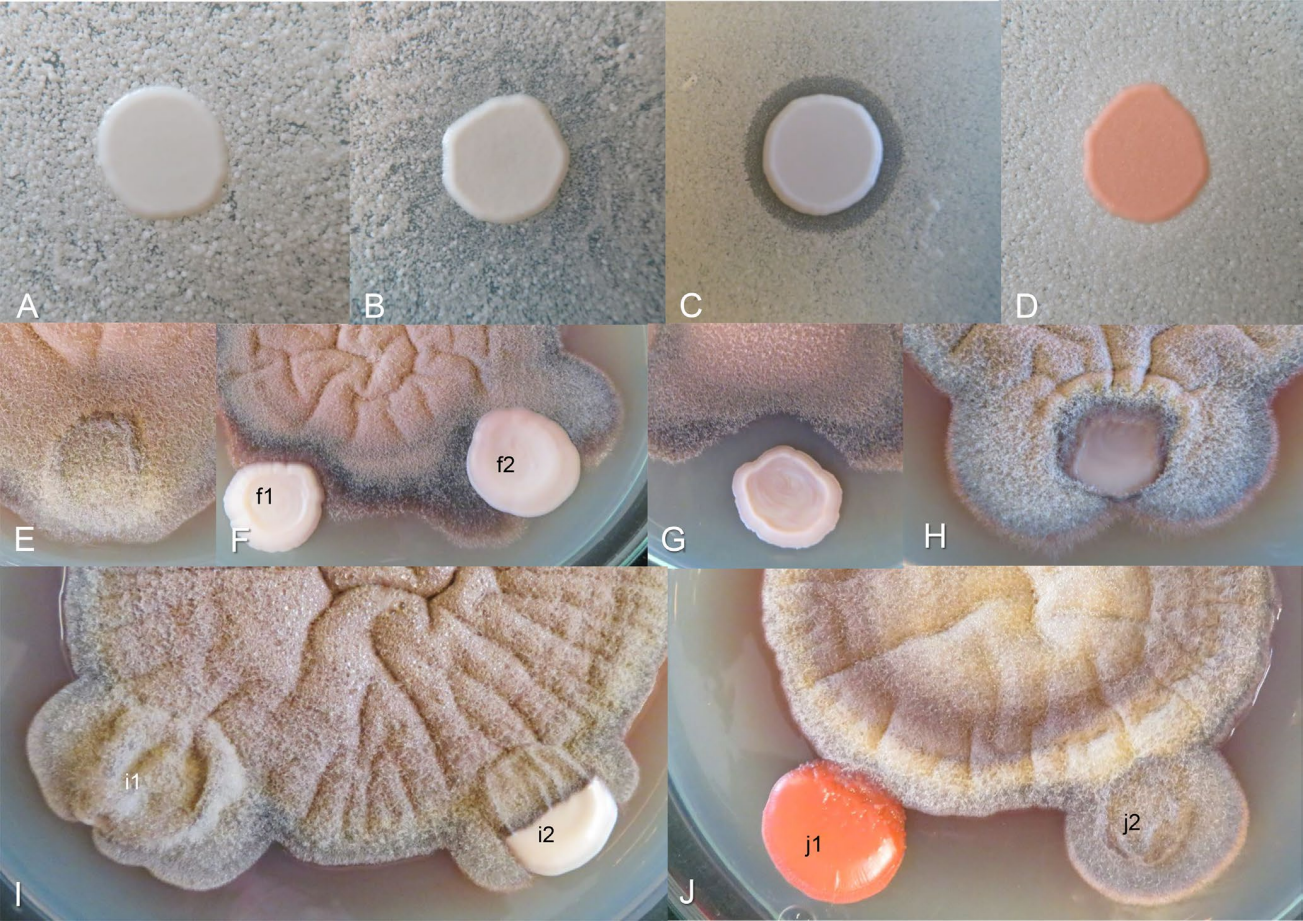
Synergistic and antagonistic effects of isolates on the growth of the test (indicator) organisms. (A) *Pseudotremella hippophaes* 11–1977. (B) *Wickerhamomyces mori* 11–1979. (C) *M. pulcherrima* 12–136. (D) *Cystofilobasidium infirmominiatum* 11–1963. (E) *Zygosaccharomyces rouxii* 12–138. (F) (f1) *M. pulcherrima* 12–12. (f2) *Debaryomyces* sp. 12–10. (G) *M. pulcherrima* 12–117. (H) *Nakazawaea holstii* 11–1976. (I) (i1) *Pseudotremella hippophaes* 12–4. (i2) *Filobasidium wieringae* 12–1. (J) (j1) *Sporobolomyces roseus* 11–1968. (j2) *Rhodotorula graminis* 11–1970. The test organisms are *Zygosaccharomyces* aff. *siamensis* 11–2106 (the lawn in A–D) and *Alternaria angustiovoidea* P101 (inoculated in the middle of the plates in E–J).

Five species showed antagonism against *Zygosaccharomyces* and 16 species reduced or inhibited the growth of the *Alternaria* mycelium. On *Zygosaccharomyces* lawn, two modes of antagonism were observed: reduction of growth intensity (a halo of thinner growth in the lawn) (Figure 5B) and inhibition of growth (a zone of no growth in the lawn) (Figure 5C). The replica‐plated prints of the former halos on fresh YEA plates grew, whereas the prints of the latter did not grow. Thus, the former mode of antagonism only reduced the growth rate of indicator cells, whereas the latter mode also killed them. A few isolates stimulated the growth of the lawn around their colonies, probably by releasing nutrients (cross‐feeding) or growth‐stimulating substances (Figure 5D).

The response of the filamentous tester to the presence of the yeast isolates was more complex (Figure 5E–J, Table 2). The majority of the isolates did not affect the growth of the mycelium (e.g., Figure 5E). At the colonies of the antagonistic isolates, two modes of inhibition were observed. The extension of the mycelium was stopped either at the edge of the yeast colony (Figure 5F) or at certain distance from it (Figure 5G). The former effect may be attributed to direct physical yeast‐hypha interactions (contact inhibition), the latter mode is rather due to inhibitors of hyphal growth secreted by the yeast cells. The colonies of a few isolates facilitated the growth of the mycelium. The stimulation was so strong that the mycelium grew faster on their colonies than on the agar medium (compare the *Sporobolomyces roseus* and *Rhodotorula graminis* colonies in Figure 5J). Remarkably, the *Metschnikowia* isolates were not uniform in antagonism either (Table S3). Dual effect, contact inhibition at the edge of the yeast colony and stimulation of the hyphal growth around the yeast colony (Figure 5F,H), was also observed.

### 3.7 Iron‐Sensitive and Iron‐Insensitive Antagonism

*Metschnikowia pulcherrima* is known to antagonise microorganisms by secreting pulcherriminic acid, a toxic iron chelator. Its secretion can easily be detected because it forms a water‐insoluble maroon‐red complex (pulcherrimin) with ferric ions. All *Metschnikowia* strains isolated in this study formed (diversely) pigmented colonies and caused pigmentation in the YEA medium supplemented with FeCl_3_ (Figure 4B,C and Table S3). Supplementation of the medium with higher concentrations of FeCl_3_ reduced the size of the pigmented halos and the inhibition zones on the *Zygosaccharomyces* lawn. Addition of iron to the medium affected neither the colour nor the antagonistic activity of the non‐*Metschnikowia* isolates.

## 4 Discussion

The success of yeast isolation from gum samples collected in geographically distant localities demonstrates that yeasts regularly occur on these exudates of sour cherry trees suffering from gumming disease. Sequencing of barcodes of the isolates revealed high taxonomic diversity. The majority of the isolates could be assigned to species but the sequences of four groups differed from the barcode sequences from all known species. These could only be assigned to genera and may represent hitherto un‐described species. Both ascomycetous and basidiomycetous yeasts were found in the samples, but the species diversity in the basidiomycetous group was somewhat higher. None of the species occurred in all samples, although *M. pulcherrima* was more abundant than any other species. However, even this yeast occurred only in 40% of the samples. From these results it can be inferred that the cherry‐tree gum does not have a characteristic yeast biota.

The sour cherry gum is a rather harsh environment for yeasts. Its high polysaccharide content could be a factor that limits yeast growth, for example by causing high osmotic pressure. Unfortunately, laboratory media containing more than 10% gum could not be prepared. So, the isolates could not be tested for osmotolerance on gum‐based media. Instead, they were tested on media supplemented with high concentrations of glucose. The ascomycetous strains coped better with higher sugar concentrations than the basidiomycetous strains, but there were exceptions. In the latter group, the osmotolerance of *Symmetrospora symmetrica* and the *Vustinia terrae* strains was comparable to that of most osmotolerant ascomycetous isolates. The former species was originally described as a moderately osmotolerant phylloplane yeast (Haelewaters et al. 2020). The latter was originally identified as soil yeast that did not grow on a medium supplemented with 50% glucose (Kachalkin et al. 2019).

All isolates grew poorly on media containing gum as the only source of nutrients. Supplementation of the medium either with glucose or with a nitrogen source slightly improved their growth, but it was necessary to supplement the medium simultaneously with both components to achieve growth intensity comparable to that observed on a standard laboratory medium. Thus, the gum is poor both in carbon and in nitrogen sources, and the isolated yeasts cannot degrade the polysaccharides of the gum to monosaccharides utilisable as carbon and energy sources.

Because of their poor growth on the non‐supplemented gum medium, the isolated yeasts are unlikely to be able to colonise the gum. It is more likely that they only adhere to the surface of the gum where the osmotic pressure is lower than inside and nutrients can also come from the environment with rain and dust. Since the young gum is soft and its surface hardens slowly during maturation, the yeasts can anchor on its surface by creating penetrating intrusions. In the laboratory tests, all ascomycetous strains but only one basidiomycetous species could penetrate the agar plate. This difference suggests that the ascomycetous yeasts can adhere to the gum surface more easily than the basidiomycetous yeasts.

The diversity of yeast populations in the samples indicates that environmental factors, such as the local microclimatic conditions, the microbiome of the surrounding vegetation and other parts of the tree, as well as its age and health status may play a significant role in shaping the composition of the gum microbiome. However, when considering the origin of the yeast colonists, the question arises whether some of them came from internal plant tissues together with the oozing material that formed the gum. As both ascomycetous and basidiomycetous yeasts have been detected within certain plants as endophytes (for recent reviews, see Doty 2013; Ling et al. 2020), this possibility deserves attention. It might be tested by simultaneously collecting surface gums and internal tissue samples from the organs of the trees on which the gums are formed.

Microorganisms living in mixed populations interact with each other and mutually shape the composition and structure of their populations (biota). In the tests of the isolates for interactions with a yeast and a mould tester (indicator) microorganism, both antagonistic and synergistic activities were observed. Morphologically, the inhibitory effect (antagonism) was manifested in several forms, indicating that inhibition could occur in several ways. In one mode, characteristic of the *Metschnikowia* isolates, clear zones of inhibition with sharp edges were formed around the colonies of the isolates in the lawn of the yeast indicator strain. These isolates also stopped the growth of the mycelium of the mould tester at a distance from their colonies. These modes of antagonism can be due to the release of a diffusible inhibitor by the yeast cells. Since the antagonism of the *Metschnikowia* isolates could be suppressed by the supplementation of the medium with a ferric salt, the released agent is most probably pulcherriminic acid. This derivative of the dipeptid cycloleucine (MacDonald 1965) is an iron chelator that inhibits the growth of other microorganisms by immobilising the free ferric ions necessary for their growth (Sipiczki 2006). The product of its reaction with ferric ions is a water‐insoluble, maroon‐red complex. The colour of the complex provided a possibility to test the rest of the isolates for pulcherriminic acid production. None of the non‐*Metschnikowia* strains formed red pigment. The *Wickerhamomyces* and the *V. terrae* colonies formed turbid zones with diffuse edges in the lawn of the yeast tester both in the presence and the absence of iron supplementation. This effect can be explained by competition for a nutrient between the isolate and the tester, or by slow releasing of an inhibitor, like a killer factor (mycocin). Numerous *W. anomalus* (for a review, see Giovati et al. 2021) and *W. silvicola* (Golubev 2015) strains have been described as killer‐toxin producers. When the filamentous fungus was the tester, an additional mode of inhibition was also observed. The mycelium reached the yeast colony but stopped growing at its edge. This mode of inhibition referred to as contact inhibition has been observed in numerous previous studies (for a review, see Sipiczki 2023), but the details of the mechanism(s) remain to be elucidated. The antifungal antagonism of yeasts adhered to the gum may contribute to the protection of the tree injuries against additional infections. Further tests should be performed to examine whether the isolates classified as antagonists in this study can also inhibit pathogenic fungi.

The opposite effect, promotion of the growth of the tester organism (synergism) was also observed around the colonies of certain yeast isolates. This phenomenon may be attributed to the release of specific nutrients (growth factors) by the cells of the isolates. The combination of antagonism (contact inhibition) and synergisms (facilitated hyphal growth) was also observed at the colonies of certain yeast isolates.

The *Metschnikowia* isolates comprised a highly heterogeneous group. Although the D1/D2 barcode sequencing assigned them to *M.* *pulcherrima*, in contrast to the clear barcode sequences of other isolates, their barcode amplicons had ambiguous nucleotides (dimorphic positions). Nucleotide ambiguity in rRNA barcode sequences is a characteristic feature of *M. pulcherrima* and is due to poor homogenisation of the repeats coding for ribosomal RNA (intragenomic diversity) (Sipiczki 2022b). Dimorphic sites were frequent in the segments of the isolates corresponding to the stems of the back‐fold D1 and D2 loops of the LSU rRNA and in the entire length of the ITS region. This difference between the two barcodes can be attributed to the different functions of the RNAs encoded by them. The D1/D2 domain is part of the Large Subunit (LSU) rRNA, which is an essential component of the ribosome. The ITS region (Internal Transcribe Sequence) located between the LSU and SSU (Small Subunit rRNA) genes does not code for a ribosomal component. It is transcribed together with the genes, but its transcript is spliced out before the SSU and SSU RNAs are incorporated in the ribosome. As the correct structure of the SSU RNA is vital for the function of the ribosome, evolution allows nucleotide substitutions only in its double‐strand loops, while the ITS region can undergo more and larger changes (for a review, see Sipiczki 2022b). The intragenomic variability of these segments causes difficulties in the taxonomic identification of strains and can lead to an overestimation of diversity in metabarcode and metagenomic analysis of environmental samples. Nevertheless, it can be used to differentiate conspecific strains and to demonstrate that their genomes are different. The isolates differed in the number and the location of ambiguous (variable) positions (intergenomic diversity), demonstrating that they had different arrays of repeats in their genomes. The RAPD patterns of the genomic DNAs of the isolates also varied. From the results of the barcode and RAPD analysis, it can be concluded that there are differences in the genome structure of the *M. pulcherrima* isolates. The tested phenotypic traits (osmotolerance, antifungal antagonism, production of pulcherriminic acid and the ability to penetrate the substrate) also showed considerable diversity. These observations are in line with previous studies that also revealed high diversity among *M. pulcherrima* strains isolated from other substrates in other locations (e.g., Sipiczki et al. 2024).

In summary, the gums of the cherry trees sampled in this study did not have a characteristic endemic yeast biota. The yeasts found in the samples can be considered the results of random contamination from the environment. The basidiomycetous strains could have come from the phylloplane, whereas the ascomycetous yeasts might have come from the bark or the fruits of the trees. Most strains of the former group belong to species that frequently occur on leaf surfaces (for a review, see Fonseca and Inácio 2006), and the majority of the latter group are strains of species colonising parts of plants in which free sugar is available. The strains persisting on the gum surface can contribute to the biological protection of the tree due to their antifungal antagonism. The most antagonistic strains belonged to *M. pulcherrima* and showed the genetic and phenotypic diversity characteristic of this species. The yeast species whose strains were found in the gum samples were originally described from different natural sources (substrates). Compared to them, the tree gum is a very different and harsh environment. It would be interesting to test their strains of different origins for the ability to cope with these conditions. The results of the tests would reveal whether the strains found on the gums are specially evolved versions of their species.

## Author Contributions

**Matthias Sipiczki:** conceptualization, investigation, funding acquisition, writing – original draft, methodology, validation, visualization, writing – review and editing, software, formal analysis, project administration, data curation, resources.

## Funding

This work was supported by Nemzeti Kutatási, Fejlesztési és Innovaciós Alap (2020‐1.1.2‐PIACI‐KFI‐2020‐00130) and University of Debrecen Programme for Scientific Publication (11111).

## Conflicts of Interest

The author declares no conflicts of interest.

## Supporting information


**Table S1:** Locations of sample collection and the list of isolates.
**Table S2:** Taxonomic identification of isolates. Only single representatives of groups showing sequence identities in the individual samples are shown.
**Table S3:** Antagonism, synergism, and invasive growth of *M. pulcherrima* isolates.
**Figure S1:** Osmotolerance of the isolated species.

## Data Availability

The data that supports the findings of this study are available in the Supporting Information of this article. All barcode sequences differing from type‐strain sequences are available in GenBank (https://www.ncbi.nlm.nih.gov/genbank/) under accession numbers listed in Table S2.
