# Supplementary material for: Yeasts on Tree Gum: Taxonomic, Genetic and Phenotypic Diversity
Source: Environ Microbiol Rep. 2026 Jul 28;18(4):e70393. doi: 10.1111/1758-2229.70393 (PMC13413228; doi:10.1111/1758-2229.70393)
Supplement: Supplementary file 1 — Table S1: Locations of sample collection and the list of isolates. Table S2: Taxonomic identification of isolates. Only single representatives of groups showing sequence identities in the individual samples are shown. Table S3: Antagonism, synergism, and invasive growth of M. pulcherrima isolates. Figure S1: Osmotolerance of the isolated species. [file EMI4-18-e70393-s001.docx]

**Supplementary Material - Tables and Figures**

**Yeasts on tree gum: taxonomic, genetic and phenotypic diversity**

Matthias Sipiczki

Department of Genetics and Applied Microbiology, University of Debrecen, Debrecen, Hungary

Institute of Horticultural Science, University of Debrecen, Debrecen, Hungary

[gecela@post.sk](mailto:gecela@post.sk); [lipovy@gmx.com](mailto:lipovy@gmx.com)

**TABLE S1** Locations of sample collection and the list of isolates

| Sample collection | | | Identification numbers of isolates kept in culture collection |
| --- | --- | --- | --- |
| Year | Geographical location | Tree identification  number |  |
| 2021 | Debrecen  Felső Józsa | 1 | 11-1963 |
|  |  | 3 | 11-1964, 11-1965 |
|  |  | 7 | 11-1966 |
|  | Debrecen  Alsó Józsa | 8 | 11-1967, 11-1968 |
|  |  | 10 | 11-1969, 11-1970 |
|  |  | 11 | 11-1971. 11-1972, 11-1973, 11-1974, 11-1975 |
|  | Zelemér | 13 | 11-1976 |
|  |  | 16 | 11-1977 |
|  | Békéscsaba,  Veľká marasť | 17 | 11-1978. 11-1979, 11-1980 |
|  |  | 18 | 11-1981, 11-1982 |
|  |  | 19 | 11-1983, 11-1984, 11-1985 |
| 2022 | Debrecen, Tégláskert | 20 | 11-1986 |
|  |  | 21 | 11-1987, 11-1988 |
|  |  | 22 | 11-1989 |
|  | Békéscsaba, cintorín Tabán | 23 | 11-1990 |
|  |  | 25 | 11-1991, 11-1992, 11-1993 |
|  |  | 27 | 11-1994, 11-1995, 11-1996 |
| 2023 | Bodaszőlő | 201 | 12-1, 12-2, 12,3 |
|  |  | 203 | 12,4, 12-5 |
|  |  | 205b | 12-6 |
|  | Zelemér | 206 | 12-7, 12-8, 12-9 |
|  |  | 208b | 12-10 |
|  | Békés,  Hatház | B2 | 12-11, 12-12, 12-13 |
|  |  | B6 | 12-14, 12-15 |
|  | Békés,  cimitir | B**7** | 12-16, 12-17 |
|  |  | B8 | 12-18 |
|  | Békéscsaba,  Malá marasť | M105 | 12-19 |
|  |  | M107 | 12-20, 12-21 |
|  |  | M116 | 12-22 |
|  | Galgaguta | M118 | 12-23, 12-24 |
|  |  | M120 | 12-25 |
|  | Vanyarc | M215 | 12-26, 12-27, 12-28 |
|  |  | M216 | 12-29 |
|  | Legénd | M218 | 12-30 |
| 2024 | Debrecen, Epreskert | 301 | 12-30/1, 12-30/2 |
|  |  | 302 | 12-117, 12-120, 12-121,  12-118, 12-119, 12-122 |
|  |  | 303 | 12-123, 12-124 |
|  | Bánk | N4/304 | 12-125, 12-126 |
|  |  | N7/307 | 12-127 |
|  | Felsőpetény | N8/308 | 12-128, 12-129 |
|  |  | N10/310 | 12-130 |
|  |  | N11/311 | 12-131, 12-133, 12-132 |
|  | Alsópetény | N12/312 | 12-134, 12-135 |
|  | Békéscsaba  Jamina | B26/326 | 12-136, 12-137 |
|  | Békéscsaba  Furiš | B29/329 | 12-138, 12-139, 12-140 |
| 2025 | Nézsa | N41/401 | 12-151, 11-152, 12-153,  12-154, 12-155 |
|  |  | N42/402 | 12-156, 12-157, 12-158 |
|  |  | N43/403 | 12-159, 12-160, 12-161 |
|  | Vanyarc | N44/404 | 12-162, 12-163 |
|  |  | N45/405 | 12-164, 12-165 |
|  |  | N46/406 | 12-166, 12-167, 12-168 |
|  | Gerendás  Hon Ulička | G407 | 12-170, 12-172, 12-169, 12-171 |
|  |  | G408 | 12-173, 12-175, 12-174 |

**TABLE S2** Taxonomic identification of isolates. Only single representatives of groups showing sequence identities in the individual samples are shown.

| Culture collection  identification number | Taxonomy (barcode sequence identity) | | | GenBank accession numbers of sequences |
| --- | --- | --- | --- | --- |
|  | Most similar type strain(s) | Sequence identity | |  |
|  |  | D1/D2 | ITS |  |
| 11-1963 | *Cystofilobasidium infirmominiatum* | 576/576 |  |  |
| 11-1964 | *Vishniacozyma*  *dimennae* | 546/571 | 457/493 | [MZ728056](https://www.ncbi.nlm.nih.gov/nuccore/MZ728056)  [MZ728057](https://www.ncbi.nlm.nih.gov/nuccore/MZ728057) |
| 11-1965 | *Vishniacozyma*  *dimennae* | 594/619 | 458/493 | [MZ728069](https://www.ncbi.nlm.nih.gov/nuccore/MZ728069)  [MZ729679](https://www.ncbi.nlm.nih.gov/nuccore/MZ729679) |
| 11-1966 | *Teunia helanensis*  *Teunia globosa*  *Teunia heritierae* | 599/620  599/620  558/566 | 600/621 | [MZ733296](https://www.ncbi.nlm.nih.gov/nuccore/MZ733296)  [MZ734329](https://www.ncbi.nlm.nih.gov/nuccore/MZ734329) |
| 11-1967 | *Pseudotremella hippophaes* | 527/529 |  | [PX129027](https://www.ncbi.nlm.nih.gov/nuccore/PX129027) |
| 11-1968 | *Sporobolomyces roseus* | 545/546 |  | [PX129028](https://www.ncbi.nlm.nih.gov/nuccore/PX129028) |
| 11-1969 | *Rhodotorula graminis* | 539/539 |  |  |
| 11-1970 | *Rhodotorula graminis* | 542/542 |  |  |
| 11-1971 | *Cystofilobasidium macerans* | 571/573 |  | [PX129030](https://www.ncbi.nlm.nih.gov/nuccore/PX129030) |
| 11-1972 | *Rhodotorula graminis* | 547/547 |  |  |
| 11-1973 | *Filobasidium wieringae* | 558/559 |  | [PX129031](https://www.ncbi.nlm.nih.gov/nuccore/PX129031) |
| 11-1974 | *Cystofilobasidium macerans* | 579/581 |  | [PX129087](https://www.ncbi.nlm.nih.gov/nuccore/PX129087) |
| 11-1975 | *Vishniakozyma tephrensis* | 556/557 |  | [PX129088](https://www.ncbi.nlm.nih.gov/nuccore/PX129088) |
| 11-1976 | *Nakazawaea holstii* | 556/556 |  |  |
| 11-1977 | *Pseudotremella hippophaes* | 533/535 |  | [PX129090](https://www.ncbi.nlm.nih.gov/nuccore/PX129090) |
| 11-1978 | *Hannaella surugaensis* | 580/581 |  | [PX129091](https://www.ncbi.nlm.nih.gov/nuccore/PX129091) |
| 11-1979 | *Wickerhamomyces mori* | 474/474 |  |  |
| 11-1980 | *Wickerhamomyces mori* | 518/518 |  |  |
| 11-1981 | *Vustinia terrae* | 538/538 |  | [MZ727613](https://www.ncbi.nlm.nih.gov/nuccore/MZ727613) |
| 11-1982 | *Meyerozyma guilliermondii* | 550/550 |  |  |
| 11-1983 | *Wickerhamomyces silvicola* | 537/539 |  | [PX129092](https://www.ncbi.nlm.nih.gov/nuccore/PX129092) |
| 11-1984 | *Nakazawaea holstii* | 524/524 |  |  |
| 11-1985 | *Nakazawaea holstii* | 552/552 |  |  |
| 11-1986 | ***Papiliotrema*** *flavescens* | 556/556 |  |  |
| 11-1987 | *Teunia helanensis*  *Teunia globosa*  *Teunia heritierae* | 600/621  600/621  558/566 | 546/572 | [MZ734329](https://www.ncbi.nlm.nih.gov/nuccore/MZ734329)  [MZ734342](https://www.ncbi.nlm.nih.gov/nuccore/MZ734342) |
| 11-1988 | *Vishniacozyma tephrensis* | 562/565 |  | [PX129093](https://www.ncbi.nlm.nih.gov/nuccore/PX129093) |
| 11-1989 | *Pseudotremella moriformis* | 591/626 | 411/480 | [MZ734343](https://www.ncbi.nlm.nih.gov/nuccore/MZ734343)  MZ736577 |
| 11-1990 | *Teunia globosa*  *Teunia helanensis*  *Teunia tronadorensis*  *Teunia heririerae* | 605/622  603/622  592/613 | 560/576 | [MZ734350](https://www.ncbi.nlm.nih.gov/nuccore/MZ734350)  [MZ734351](https://www.ncbi.nlm.nih.gov/nuccore/MZ734351) |
| 11-1991 | *Metschnikowia pulcherrima* | *** |  | [MZ734354](https://www.ncbi.nlm.nih.gov/nuccore/MZ734354) |
| 11-1992 | ***Papiliotrema*** *flavescens* | 557/557 |  |  |
| 11-1993 | ***Papiliotrema*** *flavescens* | 564/564 |  |  |
| 11-1994 | *Wickerhamomyces silvicola* | 554/554 |  |  |
| 11-1995 | *Wickerhamomyces silvicola* | 544/544 |  |  |
| 11-1996 | *Wickerhamomyces silvicola* | 537/537 |  |  |
| 12-1 | *Filobasidium wieringae* | 570/570 |  |  |
| 12-2 | *Kwoniella shivajii* | 587/587 |  |  |
| 12-4 | *Pseudotremella hippophaes* | 563/565 |  | [OR539772](https://www.ncbi.nlm.nih.gov/nuccore/OR539772) |
| 12-5 | *Pseudotremella hippophaes* | 563/565 |  | [OR539738](https://www.ncbi.nlm.nih.gov/nuccore/OR539738) |
| 12-6 | *Kwoniella shivajii* | 577/580 |  |  |
| 12-7 | *Metschnikowia pulcherrima* | *** |  | - [PX129497](https://www.ncbi.nlm.nih.gov/nuccore/PX129497) |
| 12-8 | *Nakazawaea holstii* | 547/547 |  |  |
| 12-9 | *Wickerhamomyces silvicola* | 544/546 |  |  |
| 12-10 | *Debaryomyces fabryi*  *Debaryomyces prosopidis Debaryomyces subglobosus*  *Debaryomyces hansenii*  *Debaryomyces vindobonensis* | 548/548  548/548  548/548  548/548  548/548 | 612/613  612/613  604/605  611/613  611/613 | [PX317120](https://www.ncbi.nlm.nih.gov/nuccore/PX317120) |
| 12-11 | *Metschnikowia pulcherrima* | *** |  | [PX129521](https://www.ncbi.nlm.nih.gov/nuccore/PX129521) |
| 12-12 | *Metschnikowia pulcherrima* | *** |  | [PX129526](https://www.ncbi.nlm.nih.gov/nuccore/PX129526) |
| 12-13 | *Metschnikowia pulcherrima* | *** |  | [PX129537](https://www.ncbi.nlm.nih.gov/nuccore/PX129537) |
| 12-14 | *Metschnikowia pulcherrima* | *** |  | [PX129544](https://www.ncbi.nlm.nih.gov/nuccore/PX129544) |
| 12-15 | *Metschnikowia pulcherrima* | *** |  | [PX129548](https://www.ncbi.nlm.nih.gov/nuccore/PX129548) |
| 12-16 | *Debaryomyces fabryi Debaryomyces prosopidis Debaryomyces subglobosus*  *Debaryomyces hansenii*  *Debaryomyces vindobonensis* | 545/545  545/545  545/545  545/545  545/545 | 610/611  610/611  605/606  609/611  609/611 | [PX411058](https://www.ncbi.nlm.nih.gov/nuccore/PX411058) |
| 12-17 | *Debaryomyces fabryi Debaryomyces prosopidis Debaryomyces subglobosus*  *Debaryomyces hansenii*  *Debaryomyces vindobonensis* | 586/586  586/586  586/586  586/586  586/586 | 612/613  612/613  604/605  611/613  611/613 | PX323526 |
| 12-18 | *Debaryomyces vindobonensis*  *Debaryomyces subglobosus*  *Debaryomyces prosopidis*  *Debaryomyces hansenii*  *Debaryomyces fabryi* | 587/587  587/587  587/587  587/587  587/587 | 582/584  578/579  583/584  582/584  583/584 | [PX411085](https://www.ncbi.nlm.nih.gov/nuccore/PX411085) |
| 12-19 | *Cystobasidium psychroaquaticum* | 620/621 |  | [PX129099](https://www.ncbi.nlm.nih.gov/nuccore/PX129099) |
| 12-20 | *Symmetrospora symmetrica* | 620/621 |  | [PX129100](https://www.ncbi.nlm.nih.gov/nuccore/PX129100) |
| 12-21 | *Symmetrospora symmetrica* | 611/612 |  | [PX129156](https://www.ncbi.nlm.nih.gov/nuccore/PX129156) |
| 12-22 | *Filobasidium wieringae* | 619/619 |  |  |
| 12-23 | *Filobasidium floriforme*  *Filobasidium magnum* | 585/585  585/585 |  |  |
| 12-24 | *Filobasidium floriforme*  *Filobasidium magnum* | 585/585  585/585 |  |  |
| 12-25 | *Cystobasidium slooffiae* | 584/585 |  | [PX129158](https://www.ncbi.nlm.nih.gov/nuccore/PX129158) |
| 12-26 | *Pallidophorina paarla* | 580/580 |  |  |
| 12-27 | *Pallidophorina paarla* | 563/563 |  |  |
| 12-28 | *Vishniacozyma carnescens* | 555/558 |  | [PX129163](https://www.ncbi.nlm.nih.gov/nuccore/PX129163) |
| 12-29 | *Pallidophorina paarla* | 587/587 |  |  |
| 12-30 | *Pseudotremella hippophaes* | 563/565 |  | [PX129199](https://www.ncbi.nlm.nih.gov/nuccore/PX129199) |
| 12-30/1 | *Nakazawaea holstii* | 500/500 |  |  |
| 12-30/2 | *Nakazawaea holstii* | 538/538 |  |  |
| 12-117 | *Metschnikowia pulcherrima* | *** |  | [PX129550](https://www.ncbi.nlm.nih.gov/nuccore/PX129550) |
| 12-118 | *Metschnikowia pulcherrima* | *** |  | [PX134256](https://www.ncbi.nlm.nih.gov/nuccore/PX134256) |
| 12-119 | *Danielozyma litseae*  *Gaillardinia entomophila*  *Danielozyma ontarioensis* | 474/569  459/545 | 286/350 | PV671199  PV671705 |
| 12-120 | *Gaillardinia entomophila*  *Danielozyma litseae*  *Danielozyma ontarioensis* | 383/441  389/454 | 309/383 | PV671869  PV672063 |
| 12-121 | *Gaillardinia entomophila*  *Danielozyma litseae*  *Danielozyma ontarioensis* | 451/534  465/556 | 312/381 | PV672195  PV678983 |
| 12-122 | *Danielozyma litseae*  *Gaillardinia entomophila*  *Danielozyma ontarioensis* | 471/562  453/536 | 306/378 | PV678972  PV678975 |
| 12-123 | *Metschnikowia pulcherrima* | *** |  | [PX134379](https://www.ncbi.nlm.nih.gov/nuccore/PX134379) |
| 12-124 | *Metschnikowia pulcherrima* | *** |  | [PX134973](https://www.ncbi.nlm.nih.gov/nuccore/PX134973) |
| 12-125 | *Metschnikowia pulcherrima* | *** |  | [PX134974](https://www.ncbi.nlm.nih.gov/nuccore/PX134974) |
| 12-126 | *Metschnikowia pulcherrima* | *** |  | [PX134975](https://www.ncbi.nlm.nih.gov/nuccore/PX134975) |
| 12-127 | *Metschnikowia pulcherrima* | *** |  | [PX134978](https://www.ncbi.nlm.nih.gov/nuccore/PX134978) |
| 12-128 | *Wickerhamomyces anomalus* | 545/545 |  |  |
| 12-129 | *Wickerhamomyces anomalus* | 538/538 |  |  |
| 12-130 | *Metschnikowia pulcherrima* | *** |  | [PX136267](https://www.ncbi.nlm.nih.gov/nuccore/PX136267) |
| 12-131 | *Metschnikowia pulcherrima* | *** |  | [PX136271](https://www.ncbi.nlm.nih.gov/nuccore/PX136271) |
| 12-132 | *Metschnikowia pulcherrima* | *** |  | [PX136273](https://www.ncbi.nlm.nih.gov/nuccore/PX136273) |
| 12-133 | *Metschnikowia pulcherrima* | *** |  | [PX136281](https://www.ncbi.nlm.nih.gov/nuccore/PX136281) |
| 12-134 | *Metschnikowia pulcherrima* | *** |  | [PX136289](https://www.ncbi.nlm.nih.gov/nuccore/PX136289) |
| 12-135 | *Metschnikowia pulcherrima* | *** |  | [PX136294](https://www.ncbi.nlm.nih.gov/nuccore/PX136294) |
| 12-136 | *Metschnikowia pulcherrima* | *** |  | [PX136306](https://www.ncbi.nlm.nih.gov/nuccore/PX136306) |
| 12-137 | *Metschnikowia pulcherrima* | *** |  | [PX136311](https://www.ncbi.nlm.nih.gov/nuccore/PX136311) |
| 12-138 | *Zygosaccharomyces rouxii* | 583/583 |  |  |
| 12-139 | *Lachancea thermotolerans* | 544/546 |  | [PX129227](https://www.ncbi.nlm.nih.gov/nuccore/PX129227) |
| 12-140 | *Zygosaccharomyces rouxii* | 499/500 |  | [PX129235](https://www.ncbi.nlm.nih.gov/nuccore/PX129235) |
| 12-151 | *Debaryomyces fabryi Debaryomyces prosopidis Debaryomyces subglobosus*  *Debaryomyces hansenii*  *Debaryomyces vindobonensis* | 545/545  545/545  545/545  545/545  545/545 | 616/616  616/616  608/608  615/616  615/616 | PX317127 |
| 12-152 | *Metschnikowia pulcherrima* | *** |  | [PX136790](https://www.ncbi.nlm.nih.gov/nuccore/PX136790) |
| 12-153 | *Kwoniella shivajii* | 619/620 |  | [PX129239](https://www.ncbi.nlm.nih.gov/nuccore/PX129239) |
| 12-154 | *Debaryomyces fabryi Debaryomyces prosopidis Debaryomyces subglobosus*  *Debaryomyces hansenii*  *Debaryomyces vindobonensis* | 565/566  565/566  565/566  565/566  565/566 | 611/611  611/611  607/607  610/611  610/611 | [PX411102](https://www.ncbi.nlm.nih.gov/nuccore/PX411102) |
| 12-155 | *Metschnikowia pulcherrima* | *** |  | [PX136314](https://www.ncbi.nlm.nih.gov/nuccore/PX136314) |
| 12-156 | *Metschnikowia pulcherrima* | *** |  | [PX136372](https://www.ncbi.nlm.nih.gov/nuccore/PX136372) |
| 12-157 | *Pseudotremella hippophaes* | 564/565 |  | [PX129249](https://www.ncbi.nlm.nih.gov/nuccore/PX129249) |
| 12-158 | *Metschnikowia pulcherrima* | *** |  | [PX136386](https://www.ncbi.nlm.nih.gov/nuccore/PX136386) |
| 12-159 | *Metschnikowia pulcherrima* | *** |  | [PX136634](https://www.ncbi.nlm.nih.gov/nuccore/PX136634) |
| 12-160 | *Metschnikowia pulcherrima* | *** |  | [PX136846](https://www.ncbi.nlm.nih.gov/nuccore/PX136846) |
| 12-161 | *Metschnikowia pulcherrima* | *** |  | incomplete |
| 12-162 | *Metschnikowia pulcherrima* | *** |  | incomplete |
| 12-163 | *Metschnikowia pulcherrima* | *** |  | [PX136935](https://www.ncbi.nlm.nih.gov/nuccore/PX136935) |
| 12-164 | *Metschnikowia pulcherrima* | *** |  | [PX136966](https://www.ncbi.nlm.nih.gov/nuccore/PX136966) |
| 12-165 | *Metschnikowia pulcherrima* | *** |  | [PX136969](https://www.ncbi.nlm.nih.gov/nuccore/PX136969) |
| 12-166 | *Metschnikowia pulcherrima* | *** |  | [PX136972](https://www.ncbi.nlm.nih.gov/nuccore/PX136972) |
| 12-167 | *Metschnikowia pulcherrima* | *** |  | [PX136973](https://www.ncbi.nlm.nih.gov/nuccore/PX136973) |
| 12-168 | *Metschnikowia pulcherrima* | *** |  | [PX136981](https://www.ncbi.nlm.nih.gov/nuccore/PX136981) |
| 12-168/1 | *Nakazawaea holstii* | 542/542 |  |  |
| 12-169 | *Torulaspora delbrueckii* | 591/591 |  |  |
| 12-170 | *Torulaspora delbrueckii* | 552/552 |  |  |
| 12-170/1 | *Pallidophorina paarla* | 543/543 |  |  |
| 12-171 | *Torulaspora delbrueckii* | 570/570 |  |  |
| 12-172 | *Metschnikowia pulcherrima* | *** |  | PX137568 |
| 12-173 | *Torulaspora delbrueckii* | 558/558 |  |  |
| 12-174 | *Torulaspora delbrueckii* | 594/594 |  |  |
| 12-175 | *Metschnikowia pulcherrima* | *** |  | PX137586 |

* Sequence identity was not calculated because of unambiguous nucleotides in the amplicons,

**TABLE S3** Antagonism, synergism, and invasive growth of *M. pulcherrima* isolates

| Isolate | Antagonism against | | Invasive growth  (penetration into the agar plate) |
| --- | --- | --- | --- |
|  | *Zygosaccharomyces*  (inhibition zone in mm) | *Alternaria* |  |
| 11-1991 | 0.5 | c,a | ++ |
| 12-7 | 1.25 | z | + |
| 12-11 | 0.75 | c,a | + |
| 12-12 | 0.5 | z,a | (+) |
| 12-13 | 0.75 | z | - |
| 12-14 | 1 | z | +++ |
| 12-15 | 1.25 | z | - |
| 12-117 | 1 | z | ++ |
| 12-118 | 0.75 | c,a | ++ |
| 12-123 | 1 | z,c,a | (+) |
| 12-124 | 1 | z,c,a | - |
| 12-125 | 1.25 | z | (+) |
| 12-126 | 1.75 | z,c | ++ |
| 12-127 | 1.25 | z,c,a | +++ |
| 12-130 | 1 | z | - |
| 12-131 | 1.5 | z | (+) |
| 12-132 | 0.75 | z | (+) |
| 12-133 | 1.75 | z | ++ |
| 12-134 | 1.75 | z | + |
| 12-135 | 1.25 | z,c | + |
| 12-136 | 1.5 | z,c | + |
| 12-137 | 1 | z,c | - |
| 12-152 | 2 | z | ++ |
| 12-155 | 2 | z | - |
| 12-156 | 2 | z | - |
| 12-158 | 2.25 | z | ++ |
| 12-159 | 1 | c,a | (+) |
| 12-160 | 1.75 | z,c | ++ |
| 12-161 | 1.75 | z,c | (+) |
| 12-162 | 3.25 | z | (+) |
| 12-163 | 2.75 | z | + |
| 12-164 | 2.5 | z | (+) |
| 12-165 | 1.5 | c,a | + |
| 12-166 | 2.5 | z | + |
| 12-167 | 1.5 | c | + |
| 12-168 | 2.5 | z | ++ |
| 12-172 | 3 | z | + |
| 12-175 | 2.25 | z | + |

c: contact inhibition

z. clear zone of inhibition

a: the growth of the mycelium is facilitated around the yeast colony (e.g. Fig. 5f2)


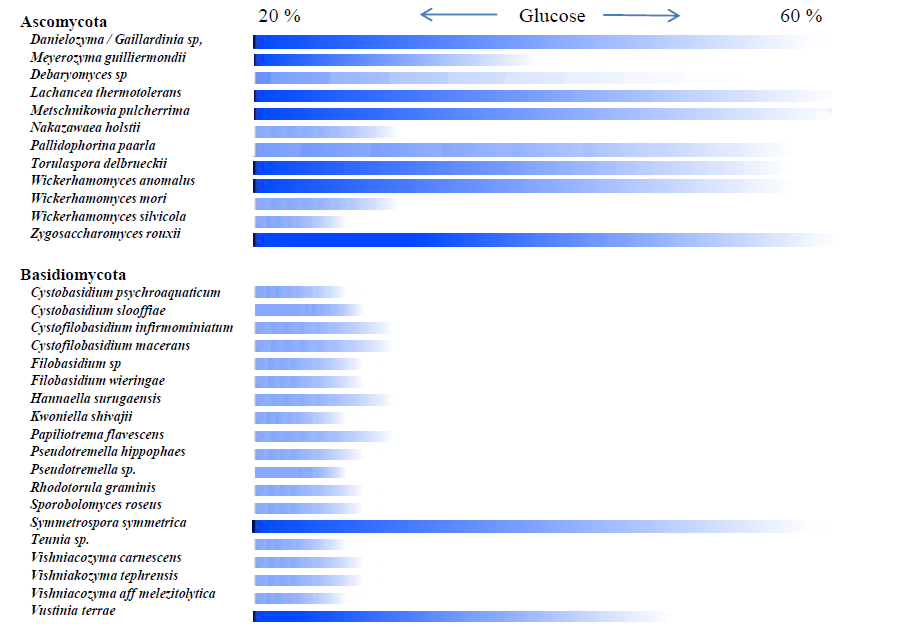


**FIGURE S1** Osmotolerance of the isolated species
